# A guide to membrane atg8ylation and autophagy with reflections on immunity

**DOI:** 10.1083/jcb.202203083

**Published:** 2022-06-14

**Authors:** Vojo Deretic, Michael Lazarou

**Affiliations:** 1 Autophagy, Inflammation and Metabolism Center of Biochemical Research Excellence, University of New Mexico Health Sciences Center, Albuquerque, NM; 2 Department of Molecular Genetics and Microbiology, University of New Mexico Health Sciences Center, Albuquerque, NM; 3 Walter and Eliza Hall Institute of Medical Research, Parkville, Victoria, Australia; 4 Department of Biochemistry and Molecular Biology, Biomedicine Discovery Institute, Monash University, Melbourne, Victoria, Australia

## Abstract

The process of membrane atg8ylation, defined herein as the conjugation of the ATG8 family of ubiquitin-like proteins to membrane lipids, is beginning to be appreciated in its broader manifestations, mechanisms, and functions. Classically, membrane atg8ylation with LC3B, one of six mammalian ATG8 family proteins, has been viewed as the hallmark of canonical autophagy, entailing the formation of characteristic double membranes in the cytoplasm. However, ATG8s are now well described as being conjugated to single membranes and, most recently, proteins. Here we propose that the atg8ylation is coopted by multiple downstream processes, one of which is canonical autophagy. We elaborate on these biological outputs, which impact metabolism, quality control, and immunity, emphasizing the context of inflammation and immunological effects. In conclusion, we propose that atg8ylation is a modification akin to ubiquitylation, and that it is utilized by different systems participating in membrane stress responses and membrane remodeling activities encompassing autophagy and beyond.

## Teaser

Deretic and Lazarou conceptualize atg8ylation as a membrane stress and remodeling response, with autophagy and nonautophagic processes as its outputs.

## Introduction

Canonical autophagy in mammalian cells is a multitasking process engaged in cytoplasmic quality control (Levine and Kroemer, 2019; Morishita and Mizushima, 2019), metabolism (Lahiri et al., 2019), and innate and adaptive immunity (Clarke and Simon, 2019; Deretic, 2021). The immune, metabolic, and quality control aspects of autophagy are intertwined in many of its physiological functions (Deretic and Kroemer, 2022). Autophagy is responsive to diverse inputs, and while the outputs may appear different, the molecular machineries involved are shared and redundantly utilized, creating sometimes unanticipated but biologically effectual overlaps. When autophagy fails, cellular, tissue, and organismal manifestations often present as dysregulated inflammation and other abnormalities. These contribute to a wide spectrum of diseases and pathological conditions reflected in animal models and human populations (Mizushima and Levine, 2020).

The term “autophagy” is often encountered in the literature as a conflation of diverse lysosomal degradative processes, including macroautophagy (herein “canonical autophagy”; Morishita and Mizushima, 2019), microautophagy (Schuck, 2020), chaperone-mediated autophagy (Bourdenx et al., 2021; Dong et al., 2021), and other systems such as formation of intralumenal vesicles and related processes in endosomes and lysosomes (Lee et al., 2020; Loi et al., 2019; Mejlvang et al., 2018). They all have a common purpose of importing substrates into membranous organelles where the sequestered substrates are typically digested. This contributes to turnover of proteins, membranes, and whole organelles; destruction of microbes or their products; and generation of metabolic intermediates during starvation (Deretic and Kroemer, 2022; Gonzalez et al., 2020; Morishita and Mizushima, 2019). There are also nondegradative outcomes of autophagy, such as secretory autophagy (Ponpuak et al., 2015), which contributes to unconventional secretion of leaderless cytosolic proteins and excretion of bulkier material from the cytoplasm.

**Figure 1.**
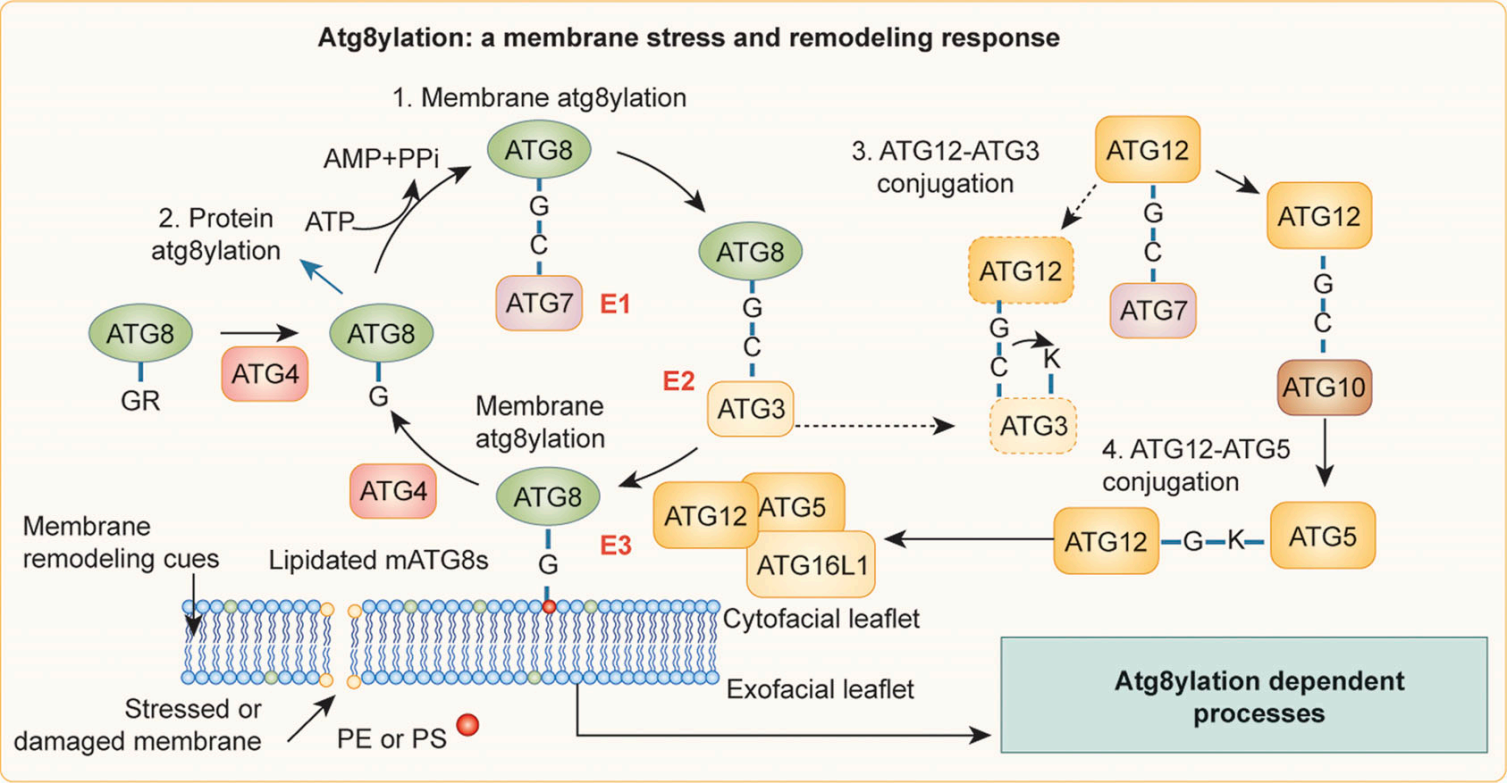
**Atg8ylation.** Membrane atg8ylation includes a ubiquitylation-like cycle of covalent modifications of membrane lipids (PE and PS) by ATG8 proteins. Note three conjugation processes (labeled 1–3): membrane Atg8ylation (driven by ATG16L1-centered E3 ligase), protein Atg8ylation, and ATG12-ATG3 conjugation as an atg8ylation-independent branch. Modified after Kumar et al. (2021b).

“Noncanonical autophagy” encompasses an assortment of autophagy-related processes akin to but different from canonical autophagy, in several cases, reflecting noncanonical activities of autophagy proteins that do not involve lysosomal degradation. These phenomena include LC3-associated phagocytosis (LAP; Sanjuan et al., 2007) and its variations (Galluzzi and Green, 2019; Ulferts et al., 2021) as well as a growing collection of diverse emerging manifestations (Goodwin et al., 2021; Guo et al., 2017a; Kumar et al., 2020; Lee et al., 2020; Leidal et al., 2020; Loi et al., 2019; Nakamura et al., 2020). A feature frequently shared by canonical and noncanonical autophagy is the engagement of mammalian ATG8 proteins (mATG8s). The mATG8 LC3B is traditionally used as an autophagy marker (Kabeya et al., 2000), although it cannot differentiate between canonical and noncanonical forms. Thus, it is necessary to reconsider what mATG8 lipidation and appearance of intracellular mATG8 puncta represent, and how we interpret them. A recent proposal is that the atg8ylation of membranes by mATG8s (Kumar et al., 2021b) represents a general membrane stress and remodeling response analogous to ubiquitylation of proteins. Under this concept, canonical autophagy (macroautophagy), LAP, and other noncanonical processes represent manifestations of the role of atg8ylation in membrane homeostasis (Kumar et al., 2021b). Here, we revisit the standard model of autophagy and related processes through the conceptual lens of atg8ylation as a general membrane stress and remodeling response (Fig. 1).

### Atg8ylation as a membrane stress and remodeling response

**Figure 2.**
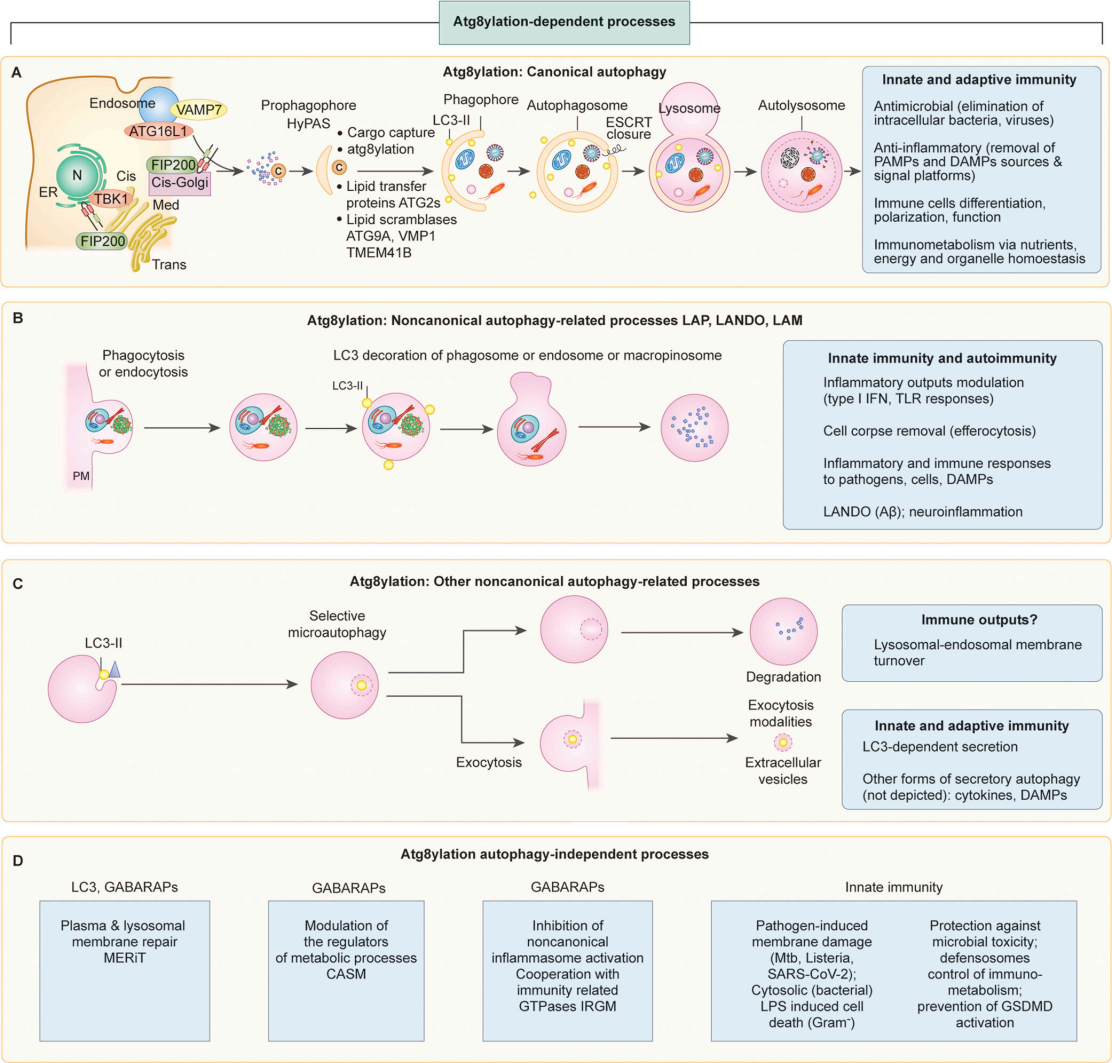
**Atg8ylation and its cell biological manifestations including canonical autophagy, noncanonical autophagy, and related nonautophagic processes. (A)** Canonical autophagy as a classic output of atg8ylation and the process of double-membrane autophagosome formation with atg8ylation-independent and atg8ylation-dependent stages. ATG8-negative prophagophore (HyPAS) is defined by fusion of FIP200^+^ early-secretory pathway/cis-Golgi–derived membrane with ATG16L1^+^ endosomal membarnes. HyPAS converts to ATG8^+^ (usually referred as LC3^+^) phagophore, which sequesters the cargo and, upon ESCRT-catalyzed membrane closure, fuses with lysosomes leading to cargo degradation. **(B and C)** Noncanonical autophagy-related processes that do not involve formation of double-membrane autophagosomes and instead rely on atg8ylation of single-membrane organelles induced in response to membrane stress or other signals requiring membrane remodeling. **(D)** Processes utilizing atg8ylation that do not include canonical or noncanonical autophagy-related processes. Boxes indicate immunological processes associated with particular atg8ylation outputs. Details in the text.

There are six main mATG8s—LC3A, LC3B, LC3C, GABARAP, GABARAPL1, and GABARAPL2/GATE16 (He et al., 2003; Weidberg et al., 2010; Xin et al., 2001)—with LC3B being universally used as the principal autophagosomal membrane marker (Kabeya et al., 2000). Whereas mATG8s are found on autophagosomes (Mizushima, 2020), autophagosomes can form without mATG8s (Nguyen et al., 2016), and autophagosome generation is initiated before their membranes become decorated with mATG8s (Kumar et al., 2021a). Importantly, mATG8s including LC3B are found on a variety of membranes other than autophagosomes (Galluzzi and Green, 2019), where they participate in diverse biological and physiological processes. This includes LAP (Sanjuan et al., 2007), its variations (Galluzzi and Green, 2019), a cluster of other related phenomena involving phagosomes or stressed endosomes (Durgan et al., 2021; Fletcher et al., 2018; Florey et al., 2015b; Florey et al., 2011; Heckmann et al., 2019; Jacquin et al., 2017; Rai et al., 2019; Ulferts et al., 2021; Xu et al., 2019), and additional processes engaging a variety of endomembranes (Goodwin et al., 2021; Guo et al., 2017a; Kumar et al., 2020; Lee et al., 2020; Leidal et al., 2020; Loi et al., 2019; Nakamura et al., 2020). The growing diversity of phenomena involving mATG8 lipidation does not easily fit the current paradigm. In a recently proposed model (Kumar et al., 2021b), membrane atg8ylation (Fig. 1, process 1) represents a generalized response to membrane stress or acts in membrane remodeling, analogous to the general role that ubiquitylation plays in tagging proteins (Hershko and Ciechanover, 1998; Mevissen and Komander, 2017). Ubiquitin and ATG8s are related in sequence and structure (Kumar et al., 2021b), and the lipidation of mATG8s, elaborated below, occurs on their C-terminal glycines, akin to the C-terminal glycine of ubiquitin (Mizushima, 2020). In addition, mATG8s can atg8ylate proteins (Fig. 1, process 2; Agrotis et al., 2019; Carosi et al., 2021; Nguyen et al., 2021). Like ubiquitylation, atg8ylation has a plethora of downstream effector outputs (Fig. 2), with autophagy being one of them. However, the full spectrum of membrane and protein atg8ylation targets, and their relatedness to membrane remodeling, is yet to be systematically explored outside of the autophagy paradigm.

Protein ubiquitylation tags them for extraction and degradation, whereas under unperturbed conditions, ubiquitylation modulates normal protein activity, localization, and interactions (Mevissen and Komander, 2017; Pohl and Dikic, 2019). Membrane atg8ylation is an analogous process acting on membranes. ATG8s are conjugated to specific membrane phospholipids, phosphatidylethanolamine (PE; Durgan et al., 2021; Ichimura et al., 2000; Lystad et al., 2019) or phosphatidylserine (PS; Durgan et al., 2021). Like ubiquitylation, atg8ylation depends on ATP and E1-like activating protein, ATG7 (Fig. 1). ATG7 is conjugated to the C-terminal glycine of Atg8s, exposed posttranslationally through cleavage mediated by ATG4 family proteases. Atg8s are next transferred to E2-like protein ATG3, and finally to the lipids PE or PS on membranes guided by an E3-like complex ATG5-ATG12/ATG16L1 (Fig. 1; Mizushima, 2020; Mizushima et al., 2011). A “sidestep” conjugation between ATG12 and ATG3 can also occur (Fig. 1, process 3; Radoshevich et al., 2010) in addition to the canonical ATG5-ATG12 conjugation (Fig. 1, process 4). Thus far, the known functions of the noncanonical ATG12-ATG3 conjugation are endosomal positioning, exosome biogenesis, and viral budding (Murrow et al., 2015). The range of biological activities of either ATG12-ATG3 conjugation (Radoshevich et al., 2010) or protein atg8ylation (Agrotis et al., 2019; Carosi et al., 2021; Nguyen et al., 2021) are yet to be fully explored.

The known processes downstream of membrane atg8ylation (Fig. 2) are individually addressed in the next section. As listed in the Fig. 2 boxes, we herein often use immunity examples to illustrate the physiological impact of atg8ylation, which broadly affects diverse aspects of metabolism, quality control, and inflammation.

### Autophagy and related processes as manifestations of atg8ylation

#### Canonical autophagy

The hallmark of canonical autophagy is the emergence of double-membrane autophagosomes in the cytoplasm (Morishita and Mizushima, 2019). Canonical autophagosomes (Morishita and Mizushima, 2019; Fig. 2 A) can capture diverse cytoplasmic cargoes that are recognized through a range of autophagy receptors (Lamark and Johansen, 2021). The autophagosomes are decorated by ATG8 family proteins, with LC3B being the most ubiquitously used marker of autophagy (Kabeya et al., 2000). Many mATG8-associated processes are termed using an LC3-based nomenclature; however, this does not exclude roles for other mATG8 family members including GABARAPs. Indeed, GABARAPs have been linked to atg8ylation-mediated activation of transcription factor EB (TFEB; Goodwin et al., 2021). We therefore note that, despite the LC3 nomenclature that is used in the field, and within this perspective, exploration into the role of each mATG8 family member is both warranted and necessary to gain a full understanding of atg8ylation-related processes. Given that studies do not always specify the LC3 subfamily member used, one may assume that it is typically LC3B. Therefore, when we refer to a study that reports a process as being decorated with or associated with LC3, be mindful that LC3B is the most likely member being discussed.

Canonical autophagy in mammals starts with the formation of a pro-phagophore via the fusion of FIP200-positive vesicles derived from the early secretory pathway (i.e., cis-Golgi) with ATG16L1-positive endosomal vesicles (Kumar et al., 2021a). The commitment to canonical autophagy is thus initiated through intermixing of two membrane sources contributed by the secretory and the endosomal pathways (Kumar et al., 2021a). Consequently, the prophagophore is referred to as a hybrid pre-autophagosomal structure (HyPAS). The HyPAS model (Fig. 2 A) of canonical autophagosome formation reconciles the two major schools of thought on the source of mammalian autophagic membranes, one being ER-centric (Axe et al., 2008; Hara et al., 2008; Hayashi-Nishino et al., 2009; Itakura and Mizushima, 2010; Itakura and Mizushima, 2011; Mizushima et al., 2011; Nishimura et al., 2017; Tooze and Yoshimori, 2010) and the other endosome-centric (Knævelsrud et al., 2013; Longatti et al., 2012; Moreau et al., 2011; Puri et al., 2013; Puri et al., 2018; Ravikumar et al., 2010; Soreng et al., 2018) and is consistent with additional studies including the reported role of the ER-Golgi intermediate compartment and coat protein II (COPII) vesicles (Ge et al., 2013; Ge et al., 2017; Ge et al., 2014). Additional contributors to autophagosomal membranes have also been reported (Hailey et al., 2010; Hamasaki et al., 2013; Nascimbeni et al., 2017; Nishida et al., 2009), which may participate at stages along the autophagosome formation pathway.

The HyPAS prophagophore is at first LC3B-negative (Kumar et al., 2021a), and it may be free of all mATG8s. While the latter notion remains to be established, it is consistent with the independence of the initial stages of autophagy from the atg8ylation system (Dalle Pezze et al., 2021; Itakura et al., 2012; Nguyen et al., 2016; Zachari et al., 2019). Atg8ylation is uncoupled from FIP200 and autophagosome formation, as observed early on with bacterial phagosomes (Kageyama et al., 2011). The LC3-negative prophagophore can in principle make initial contacts with autophagic cargo receptors, since a significant number of these receptors directly bind to FIP200 to recruit initiation complexes (Ohnstad et al., 2020; Ravenhill et al., 2019; Smith et al., 2018; Turco et al., 2019; Vargas et al., 2019). These initial encounters between the cargo and the forming autophagosomes are subsequently augmented by atg8ylation and autophagy receptor–mATG8 interactions (Padman et al., 2019; Turco et al., 2019), which coincide with conversion of the prophagophore into an atg8ylated phagophore (Kumar et al., 2021a). The phagophore enlarges via a variety of mechanisms including direct delivery of phospholipids through lipid-transfer proteins such as ATG2 (Valverde et al., 2019) as well as lipid scramblases that relax lipid asymmetry between membrane leaflets to enable lipid flow into the growing phagophore (Maeda et al., 2020; Matoba et al., 2020). The enlarged phagophore enwrapping the cargo then closes with the help of the endosomal sorting complexes required for transport (ESCRT) machinery (Takahashi et al., 2018). Closed autophagosomes deliver the captured cargo to lysosomes for degradation via autophagosome-lysosome fusion (Zhao and Zhang, 2019).

Fig. 2 A also lists the principal immunological outputs of canonical autophagy. As recently reviewed (Deretic, 2021), this includes direct microbial elimination (xenophagy), anti-inflammatory action by removing pathogen- and damage-associated molecular patterns (PAMPs and DAMPs), and development and differentiation of immune cells as well as their polarization and function. There are unique connections with immunometabolism (Deretic, 2021) because of the tight integration of canonical autophagy with the nutrient and energy sensors mammalian target of rapamycin (mTOR) and AMP-activated protein kinase (AMPK), which together determine the “posture” of key immune cells, with their populations assuming either a robust inflammatory phenotype associated with proliferation or tissue repair characteristics associated with quiescence (O’Neill et al., 2016; Saravia et al., 2020).

### Noncanonical autophagy-related processes

A number of noncanonical autophagy-related processes have been reported, and a diverse selection of newly described noncanonical trends involving mATG8s continue to emerge. The key feature of these phenomena is that they share certain but not all components with canonical autophagy and involve single instead of double membranes (Galluzzi and Green, 2019). The archetypal process in this category is LAP (Martinez et al., 2016; Sanjuan et al., 2007). Its close variations (LAP-related processes) on endosomal/phagosomal vesicles are LC3-associated endocytosis (LANDO; Heckmann et al., 2019) and LC3-associated micropinocytosis (LAM; Sonder et al., 2021; Fig. 2 B). They all exhibit the eponymous LC3 labeling but, unlike canonical autophagosomes, involve organelles with single delimiting membranes. We suggest that one may best grasp the relationship to canonical autophagy by viewing LAP and LAP-related processes as one half of the HyPAS prophagophore formation, engaging only the endosomal organelles where ATG16L1 E3 ligase resides but not the membranes coming from the early secretory pathway with FIP200 and its associated components. Consequently, FIP200 is not needed for LAP-related processes, whereas it is critical for canonical autophagy.

LAP-related processes can be induced by various stressors and membrane-damaging agents, including pharmacological agents or microbial products perturbing phagosomes and endosomes (Durgan et al., 2021; Fletcher et al., 2018; Florey et al., 2015a; Florey et al., 2011; Jacquin et al., 2017; Ulferts et al., 2021; Xu et al., 2019). The immunological roles of LAP and LAP-related processes are listed in the box in Fig. 2 B. They include anti-inflammatory activity preventing autoimmunity and lupus (Martinez et al., 2016), antimicrobial action (Kageyama et al., 2011; Martinez et al., 2015), and orderly removal of dead or dying cells and cell fragments by efferocytosis, entosis, and phagocytosis (Boada-Romero et al., 2020; Florey et al., 2011; Kim et al., 2013; Martinez et al., 2011). The downregulation of stimulator of interferon genes (STING), normally transmitting signals of ectopic presence of viral or mitochondrial dsDNA in the cytosol, via a LAP-related process (Fischer et al., 2020), has the potential to directly limit type I IFN responses, but this remains to be established. LAP may favor immune tolerance in cancer microenvironments (Cunha et al., 2018), albeit it promotes Toll-like receptor (TLR) signaling through IRF7-stimulating type I IFN response (Henault et al., 2012). In the context of neuroinflammatory diseases, LANDO clears β-amyloid aggregates and suppresses microglia activation (Heckmann et al., 2019). Of note, not all endosomes and phagosomes undergo atg8ylation, which requires a specific stress or danger signal such as described above, as well as TLR signaling upon encounter of fungal, bacterial, and microbial products known as PAMPs (Delgado et al., 2008; Sanjuan et al., 2007).

A number of other processes unrelated to LAP are manifestations of membrane atg8ylation (Fig. 2, C and D). These diverse phenomena include selective microautophagy of mammalian lysosomal membranes in response to osmotic stress or glucose starvation (Lee et al., 2020), selective removal of excess ER during recovery from ER stress via piecemeal micro-ER-phagy (Loi et al., 2019), and unconventional secretion via extracellular vesicles and secretory autophagy. Atg8ylation participates in the formation of exosomes (Guo et al., 2017a) and secretion of specific cytosolic cargo by extracellular vesicles (Leidal et al., 2020). Among the reported innate immunity functions for exosomes impacted by atg8ylation is neutralization of bacterial toxins before they can attack the host cells (Keller et al., 2020).

Atg8ylation furthermore plays a role in a type of unconventional secretion of leaderless cytosolic proteins, or excretion/extrusion of cytoplasmic material, referred to as secretory autophagy (Gerstenmaier et al., 2015; Ponpuak et al., 2015). Secretory autophagy has been implicated in the export of a key proinflammatory cytokine, IL-1β, and of the alarmin HMGB1 (Dupont et al., 2011; Karmakar et al., 2020; Kimura et al., 2017; Razani et al., 2012; Thorburn et al., 2009), albeit IL-1β exit from cells uses multiple routes (Zhang et al., 2015; Zhang et al., 2020b), including prominently a passive release of IL-1β from dying cells through gasdermin pores on the plasma membrane during pyroptosis (Broz et al., 2020; Evavold et al., 2018). Atg8ylation does not always protect the host: for example, it has been coopted by influenza A to promote its filamentous mode of budding from the plasma membrane (Beale et al., 2014).

In summary, atg8ylation participates in phenomena historically referred to as noncanonical autophagy but functionally representing a range of membrane stress responses and membrane remodeling processes. This diversity is mirrored by the equally diverse immunological and anti-inflammatory outputs of atg8ylation further discussed below. Despite atg8ylation being linked to noncanonical processes, how exactly mATG8s are functioning during many of these processes remains to be fully explored. It is likely that atg8ylation is involved in the formation/expansion of membrane vesicles, but mATG8s may also recruit factors through LC3 interaction region (LIR)/GABARAP interaction motif (GIM), thereby acting as scaffolds for protein complexes that play roles in signaling. In addition, atg8ylation may play an adaptor role to recruit factors within the lumen of vesicles. In the next section, we cover some of the recent advances in the signaling roles of membrane atg8ylation.

### Atg8ylation and signaling

**Figure 3.**
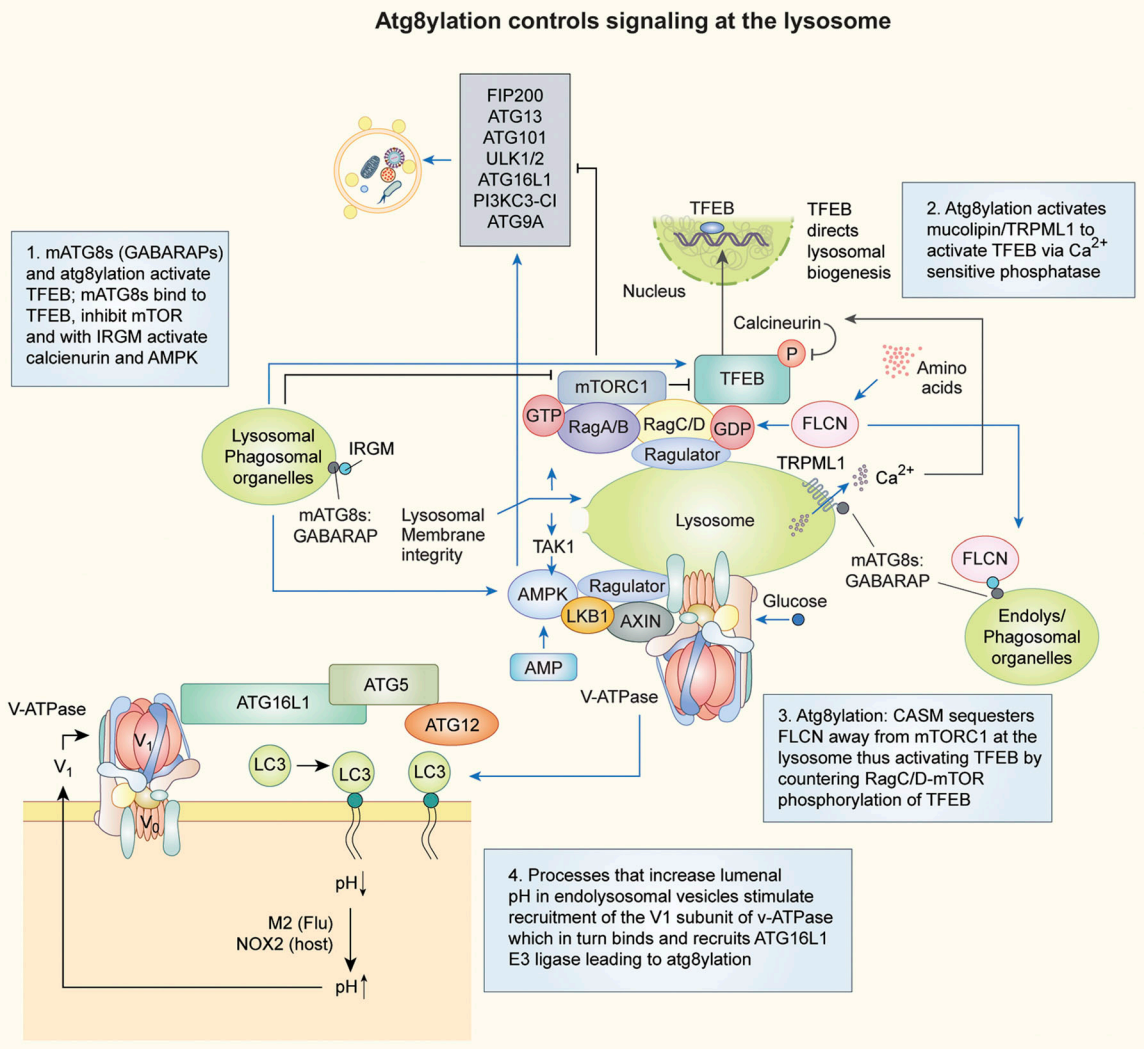
**Specific examples and circuitry of how atg8ylation controls different signaling and stress response processes at the lysosome.** Note that both mTOR and AMPK as well as their regulatory elements are localized at the lysosome. AMPK positively regulates canonical autophagy, whereas mTOR negatively regulates this atg8ylation-associated process. Boxes 1–4 describe four of the expanding list of autophagy-independent atg8ylation-dependent processes. This includes control of AMPK, mTOR, and TFEB by mATG8s and atg8ylation (Boxes 1–3). Box 4–associated schematic depicts how increase in lumenal pH (phagosomes, organelles of the endolysosomal network) by the action of the influenza viroporin M2 that acts as an H^+^ channel or proton scavenging during superoxide production induce membrane atg8ylation (LC3 shown as an example of LAP and LAP-related processes). This occurs due to the increased recruitment of ATG16L1 via its direct binding to the V1 subunit of vacuolar H^+^-ATPase, upon elevated V_1_V_0_ assembly on membranes in response to neutralization of the lumenal pH. Further details in the text.

Membrane atg8ylation can affect multiple signaling systems and therefore functions beyond the proposed direct physical role in membrane remodeling during the formation of canonical autophagosomes (Maruyama et al., 2021) and noncanonical LAP-like structures (Galluzzi and Green, 2019). The atg8ylation that takes place during canonical autophagy is the most understood (Kabeya et al., 2000). During PINK1/Parkin mitophagy, atg8ylation can serve as an amplification signal by increasing the concentration of autophagy machineries on phagophores (Padman et al., 2019). Although PINK1/Parkin mitophagy is largely independent of mTOR and AMPK signaling (Vargas et al., 2019), most forms of canonical autophagy depend on these principal regulators of cellular metabolism (Deretic and Kroemer, 2022). Localized at least in part to lysosomes (Fig. 3), mTOR inhibits (whereas AMPK activates) protein complexes controlling autophagosome biogenesis. Lysosomes are also the site where these systems, along with phosphatases such as calcineurin, control TFEB, a key regulator of lysosomal biogenesis, with functional lysosomes being essential for the completion of canonical autophagy and other roles (Ballabio and Bonifacino, 2020).

A specific subset of mATG8s, GABARAPs, along with their atg8ylation onto membranes, help activate TFEB under different conditions (Goodwin et al., 2021; Kumar et al., 2020; Nakamura et al., 2020; Fig. 3) as follows. (1) GABARAPs bind to TFEB (Fig. 3, box 1), inhibit mTOR, and together with immunity-related GTPase M (IRGM), which stabilizes AMPK (Chauhan et al., 2015a), activate calcineurin phosphatase PPP3CB (Kumar et al., 2020). PPP3CB in turn dephosphorylates TFEB, resulting in its nuclear translocation and transcriptional activation of the lysosomal biogenesis program (Medina et al., 2015). (2) During lysosomal damage, atg8ylation stimulates the Ca^2+^ channel mucolipin/TRPML1 to activate TFEB via a phosphatase different from calcineurin (Nakamura et al., 2020; Fig. 3, box 2), possibly PP2A (Hasegawa et al., 2022). (3) Membrane atg8ylation at various locations in the cell indirectly affects the activation state of mTOR on the lysosome (Goodwin et al., 2021; Fig. 3, box 3). At the lysosome, mTOR is controlled by a set of Rag GTPases and folliculin (FLCN), which acts on RagC/D to maintain TFEB phosphorylated by mTOR (Napolitano et al., 2020). Atg8ylation with GABARAPs elsewhere in the cell sequesters FLCN away from Rags-mTORC1 on the lysosome, resulting in TFEB activation. Such atg8ylation of membranes at remote sites includes canonical autophagy (e.g., mitophagy), phagocytosis of bacteria and associated LAP, and pharmacological stress (Goodwin et al., 2021).

How is membrane atg8ylation induced on endolysosomal and phagosomal vesicles? Agents that deacidify/increase lumenal pH in endolysosomal vesicles (Ulferts et al., 2021) stimulate recruitment of the V_1_ subunit of v-ATPase, which in turn recruits ATG16L1 E3 ligase (Xu et al., 2019; Fig. 3, box 4). This leads to direct membrane atg8ylation without formation of autophagosomes (Fischer et al., 2020; Hooper and Florey, 2021 *Preprint*; Ulferts et al., 2021). Of relevance for the immunological subplot of this article, many of the known physiological examples of membrane atg8ylation involve interactions of host cells with microbes. This includes *Salmonella* (Xu et al., 2019) and viruses such as influenza (Ulferts et al., 2021), response to microbial or ectopic dsDNA in the cytosol (Fischer et al., 2020), and activation of NADPH oxidase (Hooper and Florey, 2021 *Preprint*) by phagocytic cells as they kill microbes (Nauseef, 2019).

In addition to lysosomal membrane atg8ylation, extensive physical damage of these membranes eventually elicits canonical autophagy to remove excessively damaged lysosomes by lysophagy. Damage of endolysosomal organelles occurs physiologically, e.g., during exposure to exogenous and endogenous agents including biologically active crystals of silica, monosodium urate, and cholesterol (Maejima et al., 2013; Razani et al., 2012; Schroder and Tschopp, 2010), proteopathic fibrils or amyloid (Heneka et al., 2013; Masters et al., 2010; Papadopoulos et al., 2017; Parry et al., 2015), TRAIL-signaling induced lysosomal permeabilization (Werneburg et al., 2007), microbes including bacteria *Salmonella* and *Mycobacterium tuberculosis* (Chauhan et al., 2016; Fujita et al., 2013; Jia et al., 2018; Thurston et al., 2012; Watson et al., 2012), and coronaviruses including SARS-CoV-2 (Ghosh et al., 2020; Yue et al., 2018). A breach in lysosomal membrane integrity is subject to repair by ESCRTs (Jia et al., 2020b; Radulovic et al., 2018; Skowyra et al., 2018) or removal by canonical autophagy (Fujita et al., 2013; Jia et al., 2018; Jia et al., 2020b; Jia et al., 2020c; Maejima et al., 2013). Atg8ylation is a part of the canonical autophagy of excessively damaged lysosomes (lysophagy) set in motion by the inactivation of mTOR and the activation of AMPK (Fig. 3). The latter stages of lysophagy are aided by systems enabling selective autophagy of damaged lysosomes: the recognition of exposed lumenal glycans by ubiquitin E3 ligases and protein ubiquitination (Yoshida et al., 2017), ubiquitin remodeling (Papadopoulos et al., 2017), atg8ylation of autophagosomal membrane (Maejima et al., 2013), and engagement of selective autophagy receptors possessing LIRs (Lamark and Johansen, 2021) and ubiquitin-binding or galectin-binding capabilities, including p62/SQSTM1 (Papadopoulos et al., 2017), TRIM16 (Chauhan et al., 2016), and TAX1BP1, along with its interactors TBK1 and FIP200 (Eapen et al., 2021).

In conclusion, membrane atg8ylation, whether occurring on a single membrane delimiting an organelle of the endocytic pathway or on the double membrane of the canonical autophagosome, plays a significant role in upstream signaling and downstream effector processes.

### Atg8ylation and autophagy integrate innate immunity, metabolism, and quality control signals

**Figure 4.**
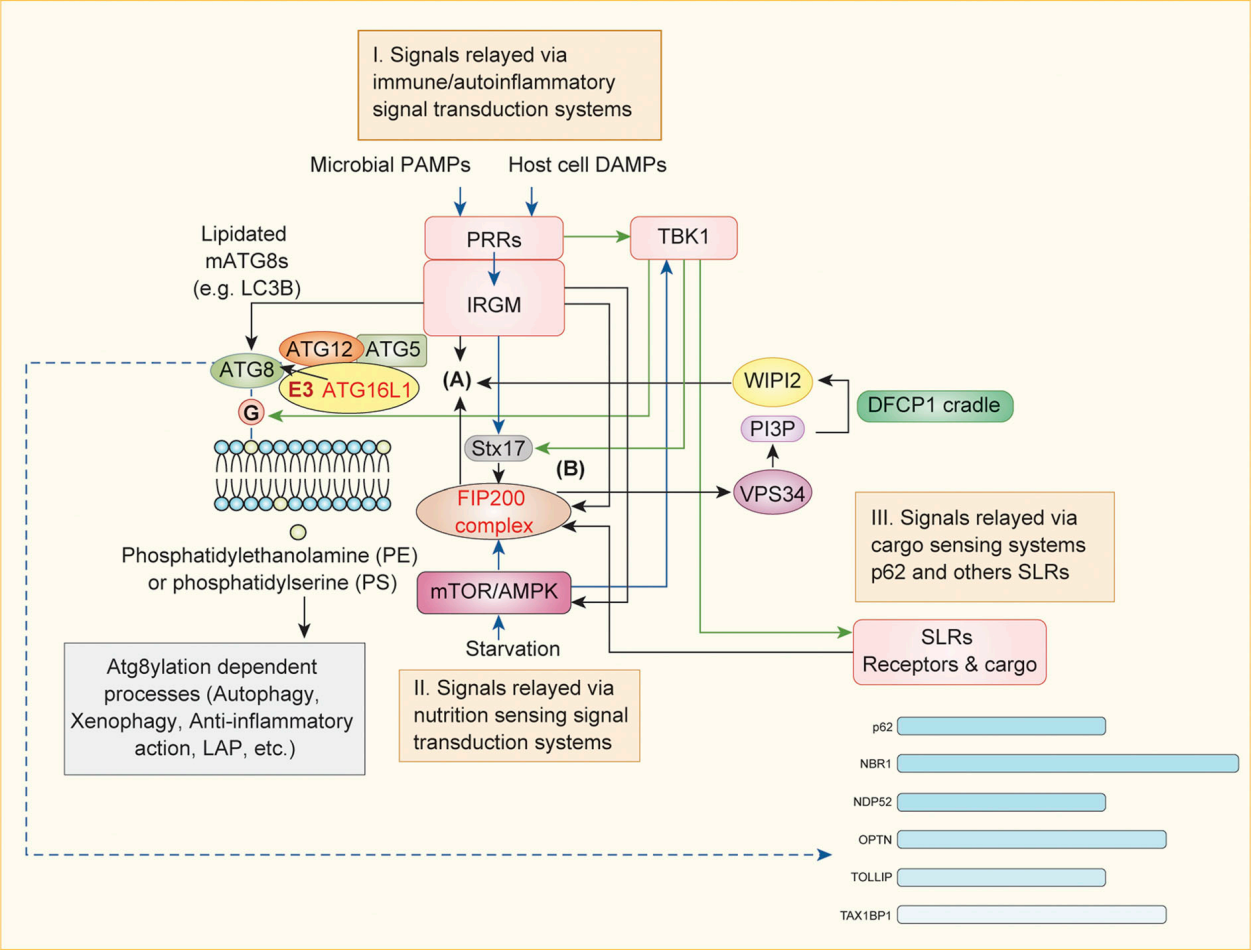
**Signaling inputs into the systems regulating atg8ylation and canonical autophagy.** To underscore that atg8ylation apparatus can act independently of canonical autophagy, the system is split into two parts (A and B). Atg8ylation E3 ligase centered upon ATG16L1 (A; see Fig. 1) and the FIP200 complex (B). For canonical autophagy, A and B come together (see Fig. 2). Three types of major inputs affecting components A or B or both are in peach-colored boxes (I–III) and fall in three categories: immune signals (I), signals coming from selective autophagy receptors (II), and metabolic signals (III). Immune signals are collected via PRRs assisted in many cases by immunity-related GTPases such as IRGM and often (but not exclusively) transduced via TBK1 to several components controlling atg8ylation and canonical autophagy apparatus. Cargo recognition by SLRs relays cargo capture, whereas SLRs can associate with FIP200 and in turn receive further signals from TBK1. Note that the ATG16L1 E3 ligase participates in FIP200 complex–independent standalone atg8ylation processes such as LAP and others (categorized in Fig. 1). Details in the text.

The biological outputs of autophagy and its noncanonical forms fall into three categories: metabolic, quality control, and immune. These signals converge on the same key components in the context of membrane atg8ylation or canonical autophagy (Fig. 4). As discussed earlier, one may consider the canonical autophagy systems split into two halves coming together during the formation of autophagosomes (Axe et al., 2008; Fujita et al., 2013; Gammoh et al., 2013; Hara et al., 2008; Hayashi-Nishino et al., 2009; Itakura and Mizushima, 2010; Itakura and Mizushima, 2011; Kumar et al., 2021a; Mizushima et al., 2011; Nishimura et al., 2013; Nishimura et al., 2017; Tooze and Yoshimori, 2010): (1) the atg8ylation machinery centered on ATG16L1 as the key part of the atg8ylation E3 ligase (Fig. 4; component A); and (2) the FIP200 complex (Fig. 4; component B), whose participation distinguishes canonical autophagy from noncanonical autophagy-related processes. Three categories of signals converge on these components (Fig. 4): (a) inputs relayed by the immune and autoinflammatory signal transducing systems (Fig. 4, box 1); (b) inputs relayed by the nutrition-sensing signal transducing systems (Fig. 4, box 2); and (c) signals relayed from selective autophagy cargo receptors such as sequestosome 1/p62-like receptors (SLRs) and others (Fig. 4, box 3).

#### Pattern recognition receptors (PRRs)

Among the innate immunity signals feeding into component A, centered on the atg8ylation E3 ligase ATG16L1 (Fig. 4), are microbial PAMPs or endogenous irritants and misplaced macromolecules referred to as DAMPs. The presence of PAMPs or DAMPs is recognized by innate immunity receptors collectively termed PRRs and relayed to the atg8ylation apparatus with or without canonical autophagy (Fig. 4). Nearly all classes of PRR act to stimulate atg8ylation. This includes TLRs (Delgado et al., 2008; Sanjuan et al., 2007) and NOD-like receptors (NLRs; Chauhan et al., 2015a; Cooney et al., 2010; Travassos et al., 2010), which recognize a wide variety of microbial products and induce atg8ylation. RIG-I–like receptors (RLRs) recognize short viral double-stranded RNA (dsRNA) and long dsRNA and detect absence of modifications in the 5′ capped mRNA, whereas their adaptor mitochondrial antiviral signaling protein (MAVS) binds mATG8s and possibly participates in mitophagy (Sun et al., 2016). Of note, MAVS activates TBK1 (Liu et al., 2015), which in turn stimulates autophagy. Atg8ylation and its manifestations respond to cytosolic dsDNA recognized by the cGAS-STING system (Gui et al., 2019) and possibly to cytosolic dsRNA recognized by cGAS-like receptors, which also engage STING (Slavik et al., 2021). STING has been implicated in membrane atg8ylation (Fischer et al., 2020; Gui et al., 2019; Liu et al., 2019), canonical autophagy (Moretti et al., 2017; Rong et al., 2022; Zhang et al., 2020b), and noncanonical autophagy-related processes (Gui et al., 2019; Liu et al., 2019), many with innate immunity and other outputs (Gui et al., 2019; Moretti et al., 2017; Watson et al., 2015; Watson et al., 2012; Yamashiro et al., 2020) including neuroinflammation (Sliter et al., 2018). Several of these systems interact with ATG16L1, either directly (Cooney et al., 2010; Travassos et al., 2010) or with the help of an immunity-related small GTPase, IRGM (Chauhan et al., 2015a; Singh et al., 2006).

#### TBK1

TBK1 can regulate atg8ylation-associated activities and autophagy (Kumar et al., 2021a; Pilli et al., 2012; Thurston et al., 2009; Wild et al., 2011). It is a key innate immunity kinase (Fitzgerald et al., 2003) best known to immunologists for transducing PAMP and DAMP signals via cGAS-STING, TLR4-TRIF, and RIG-I-MAVS (Liu et al., 2015). As an example of integration of various signals, TBK1 is linked to metabolic signaling via AMPK (Zhao et al., 2018) and mTOR (Antonia et al., 2019; Zhu et al., 2019; Fig. 4). In the processes of atg8ylation and autophagy, TBK1 acts in a multitude of ways (Fig. 4). First, TBK1 phosphorylates STX17 at S202 (Kumar et al., 2019; Rong et al., 2022), which in turn affects FIP200 complexes and catalyzes the formation of the autophagic prophagophore HyPAS (Kumar et al., 2021a). Second, TBK1 activates the selective autophagy by directly phosphorylating SLRs: OPTN (Wild et al., 2011), p62/SQSTM1 (Pilli et al., 2012) and NDP52 and TAX1BP1 (Richter et al., 2016), in addition to forming physical complexes with NDP52 (Ravenhill et al., 2019; Thurston et al., 2009; Vargas et al., 2019). Third, and possibly critical for atg8ylation, TBK1 stabilizes lipidated LC3C and GABARAPL2 by phosphorylating them and protecting them from delipidation by ATG4 isoforms (Herhaus et al., 2020). In these functions, TBK1 is involved in antibacterial autophagy (Pilli et al., 2012; Thurston et al., 2009; Wild et al., 2011), antiviral autophagy (Sparrer et al., 2017; Yamashiro et al., 2020), mitophagy (Heo et al., 2015; Lazarou et al., 2015; Moore and Holzbaur, 2016; Vargas et al., 2019), and even ER-phagy when it is provoked by bacterial PAMPs (Moretti et al., 2017).

#### IRGM

Human IRGM (Fig. 4) and its murine paralogs interact with mATG8s (Kumar et al., 2020), leading to multiple effector outputs. These include recruitment of STX17 (Kumar et al., 2018) as a component of the HyPAS fusion apparatus (Kumar et al., 2019) leading to the formation of autophagic prophagophores (Kumar et al., 2021a), as well as inhibition of mTOR (Kumar et al., 2020) and stabilization of AMPK (Chauhan et al., 2015a), which regulate the FIP200 complex en route to canonical autophagosomes (Kumar et al., 2021a). IRGM and its paralogs bind to both ATG16L1 and mATG8s and may present mATG8s to the ATG16L1 E3 ligase, leading to atg8ylation. Among the IRGM-dependent immunological effects is the protection of cells from excessive cGAS-STING and RIG-I-MAVS signaling (Jena et al., 2020), from excessive activation of the canonical (Mehto et al., 2019) and noncanonical inflammasomes, and from ensuing pyroptosis during bacterial invasion (Eren et al., 2020; Finethy et al., 2020).

#### Signals relayed via cargo sensing by selective autophagy receptors

Multiple SLRs (OPTN1, NDP52, SQSTM1, TAX1BP1, and NBR1) and other autophagy receptors recognize cargo and make initial contacts with ubiquitinated targets, followed by phagophore formation on the cargo surface (Lazarou et al., 2015; Ohnstad et al., 2020; Ravenhill et al., 2019; Smith et al., 2018; Turco et al., 2019; Vargas et al., 2019). These receptors transduce cargo recognition signals to the FIP200 half of the canonical autophagy apparatus (Fig. 4). These signals join those flowing from TBK1, an interactor and a modifier of SLRs in their antimicrobial roles (Pilli et al., 2012; Sparrer et al., 2017; Thurston et al., 2009; Wild et al., 2011) and in their role of removing mitochondria as sources of DAMPs (Heo et al., 2015; Lazarou et al., 2015; Sliter et al., 2018). In addition to SLRs, a whole group known as tripartite motif proteins (TRIMs) participate in autophagy (Kimura et al., 2016), in part by functioning as selective autophagy receptors (Mandell et al., 2014; Kimura et al., 2015; Jena et al., 2018; Di Rienzo et al., 2020; Saha et al., 2022), and as regulators of type I IFN responses (Versteeg et al., 2013). SLRs and TRIMs interact with components from both halves of the autophagic apparatus defined by FIP200 and ATG16L1 (Fig. 4, components A and B).

#### Metabolic signals

AMPK and mTOR are key activators of canonical autophagy at times of nutritional, energy, and growth factor limitations (Gonzalez et al., 2020; Morishita and Mizushima, 2019). Signals coming from macro- and micronutrients including intracellular levels of amino acids, glucose, acetyl coenzyme A (via acetylation and deacetylation of proteins), free fatty acids, etc., influence various components of the autophagy apparatus (Deretic and Kroemer, 2022). These inputs engage both halves of the canonical autophagy pathway: (a) the atg8ylation E3 ligase ATG16L1 half (Fig. 4, component A; Alsaadi et al., 2019) and (b) the FIP200 half (Fig. 4, component B; Egan et al., 2011; Ganley et al., 2009; Hosokawa et al., 2009; Jung et al., 2009; Kim et al., 2011). AMPK and mTOR are in turn affected by atg8ylation in a feedback loop (Goodwin et al., 2021; Kumar et al., 2020; Nakamura et al., 2020).

AMPK and mTOR functionally interact with the v-ATPase at the lysosome (Fig. 3; Eaton et al., 2021). This is important for components of the canonical autophagy pathway, and for atg8ylation processes in general via the ATG16L1 recruitment to and association with the V_1_ subunit of the v-ATPase (Hooper and Florey, 2021 *Preprint*; Ulferts et al., 2021; Xu et al., 2019; Fig. 3, bottom left). The v-ATPase is a potent hub for integration of signals leading to canonical autophagy and other atg8ylation manifestations.

There is ample evidence for integration of metabolic and immune inputs in controlling atg8ylation and autophagic processes. AMPK, mTOR, and ULK1 are modulated by immune signal transducers IRGM (Chauhan et al., 2015a; Kumar et al., 2020), TBK1 (Antonia et al., 2019; Zhao et al., 2018; Zhu et al., 2019), and NOD2 and RIPK2 (Lupfer et al., 2013). This is also reflected in many overlaps of canonical and noncanonical autophagic processes (Deretic, 2021; Riffelmacher et al., 2018) with immunometabolism (Brady et al., 2018; O’Neill et al., 2016; Saravia et al., 2020).

### Anti-inflammatory functions of atg8ylation and autophagy

**Figure 5.**
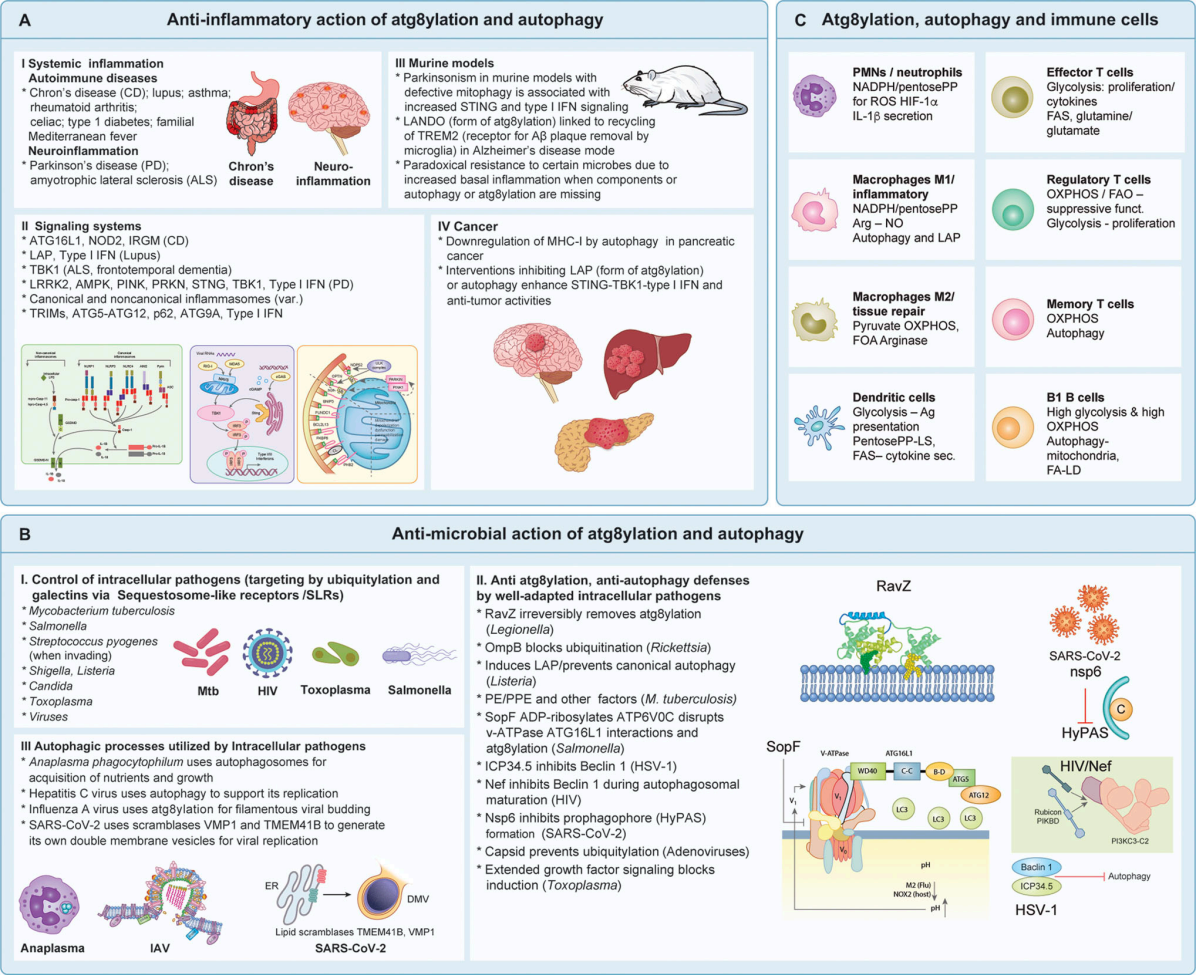
**Atg8ylation and autophagy roles in innate immunity and immune cells. (A)** Summary of anti-inflammatory action in disease contexts and animal models, and illustrations of proinflammatory signaling platforms targeted by atg8ylation or autophagy. **(B)** Direct antimicrobial action of atg8ylation and autophagy and microbial adaptations to counter or utilize atg8ylation or autophagy. **(C)** Roles of atg8ylation and autophagy in different types of immune cells, including homeostasis and immunometabolism. M1 and M2 refer to classically and alternatively activated macrophages. Details are given in the text.

Deficiencies in canonical autophagy and noncanonical autophagy-related processes, along with the emerging concept of membrane atg8ylation, correlate with inflammatory or autoimmune disorders as detailed in recent reviews (Deretic, 2021; Galluzzi and Green, 2019; Youle, 2019). This includes (Fig. 5 A, box I) autoimmune diseases and conditions with systemic inflammation such as Crohn’s disease (CD), lupus, asthma, rheumatoid arthritis, celiac disease, type 1 diabetes mellitus, and familial Mediterranean fever. These relationships extend to neurological disorders with inflammatory components, including Parkinson’s disease (PD), amyotrophic lateral sclerosis (ALS), and frontotemporal dementia. We refer the reader to a recent review for details (Deretic, 2021).

Some of the earliest genomewide association studies of any human disease (Consortium, 2007) revealed connections between polymorphisms in the genes encoding ATG16L1 and IRGM with the increased risk for CD. ATG16L1 and IRGM form a tripartite complex with NOD2 (Chauhan et al., 2015b), a familial CD predisposition locus (Horowitz et al., 2021; Fig. 5 A, box II). This complex participates in anti-inflammatory processes via several mechanisms that involve canonical or noncanonical autophagy (Jena et al., 2018; Jena et al., 2020; Mehto et al., 2019; Singh et al., 2006), both of which are downstream outputs of atg8ylation.

Metabolic inputs and innate immunity systems may integrate with autophagic and other atg8ylation processes in the context of neurodegenerative diseases (Fig. 5 A, box II; Youle, 2019). TBK1 is a frequent site of mutations in ALS (Ahmad et al., 2016). TBK1 can be controlled by AMPK and ULK1, whereas TBK1 directly phosphorylates mTOR and activates both mTORC1 and mTORC2 in response to growth factors or PAMP-PRR signaling (Bodur et al., 2018; Tooley et al., 2021). LRRK2, a protein kinase affecting autophagy (Gomez-Suaga et al., 2012) and axonal transport of autophagosomes (Boecker et al., 2021), is commonly associated with familial PD as well as CD, leprosy, and certain cancers. LRRK2 positively regulates AMPK (Usmani et al., 2021). Metabolic changes have been observed in astrocytes derived from PD patients carrying a common LRRK2 mutation (Sonninen et al., 2020). It is worth noting that LRRK2 operates, among other compartments, within the endolysosomal system where various manifestations of atg8ylation take place. PD has been associated with inflammation, including elevated cytokines in both serum and cerebrospinal fluid (Dzamko et al., 2015). In mouse models, the regulators of mitophagy PINK1 and Parkin (Youle, 2019) have been difficult to connect with PD, likely owing to the stress-responsive nature of PINK1 and Parkin and the lack of stress exposure to lab mice. However, under conditions promoting mitochondrial stress and inflammation, manifestations of parkinsonism could be detected (Sliter et al., 2018), including during an extended aging period (Noda et al., 2020). The PD phenotype could be rescued by manipulating STING (I199N missense mutant allele of the murine Sting gene, *Tmem173*) in *Prkn*^*−/−*^ or *Pink1*^*−/−*^ mice (Sliter et al., 2018). These findings help connect the dots between failure in mitophagy (which includes atg8ylation), mitochondrial DNA release, cytosolic DNA sensing systems, and inflammation (Borsche et al., 2020; McArthur et al., 2018; Sliter et al., 2018; White et al., 2014). Although mitochondria are the major metabolic factories of the cell, here they are implicated as a source of DNA acting as a DAMP (Borsche et al., 2020), stimulating STING-TBK1 signaling to elicit a type I IFN response (Sliter et al., 2018).

Inflammasomes are cytosolic signaling complexes activated by PAMPs and DAMPs and can be canonical (Lamkanfi and Dixit, 2014) or noncanonical (Broz et al., 2020; Fig. 5 A, box II). They proteolytically activate IL-1β and lead to its secretion along with other proinflammatory cytokines. A canonical inflammasome is centered on ASC (apoptosis-associated speck-like protein containing a CARD), pro-caspase 1, and microbial product sensors (Lamkanfi and Dixit, 2014), whereas noncanonical inflammasomes directly recognize cytosolic LPS and activate murine caspase-11 or human caspase-4 or -5, resulting in proteolytic processing of gasdermin D, causing pyroptosis (Broz et al., 2020). Autophagy or atg8ylation indirectly suppresses canonical inflammasome activation by reducing sources of DAMPs and reactive oxygen species (ROS; Nakahira et al., 2011; Sumpter et al., 2016; Zhou et al., 2011) or by downregulating inflammasome components (Kimura et al., 2015; Liu et al., 2016; Mehto et al., 2019; Shi et al., 2012). IRGM and mATG8s, likely via atg8ylation, deny LPS access to the cytosol associated with bacterial entry, thus dampening noncanonical inflammasome activation and pyroptosis (Eren et al., 2020; Finethy et al., 2020).

Protein complexes that stimulate type I IFN production are affected by atg8ylation components or the whole autophagy pathway as one of the most studied downstream outputs of atg8ylation (Fig. 5 A, box II). The ATG5–ATG12 conjugate, a key part of the atg8ylation E3 ligase ATG16L1 complex, inhibits RIG-I (Jounai et al., 2007). ATG9A negatively controls trafficking of the ER-associated STING and inhibits activation of TBK1 (Saitoh et al., 2009). In the absence of effective mitophagy, RIG-I-like-MAVS signaling is increased due to high MAVS levels and ROS emanating from depolarized mitochondria (Tal et al., 2009). TRIM21 targets both IKKβ (Niida et al., 2010) and IRF3 (Kimura et al., 2015) for degradation ascribed to autophagy. cGAS (Chen et al., 2016), RIG-I, and TLR3 (Jena et al., 2020), as well as STING (Prabakaran et al., 2018), seem to be attenuated through autophagy to reduce type I IFN signaling. Targeting by mATG8s and downregulation of immune signaling proteins extends to TAB1/TAB2-TAK1, with effects on downstream IKK complexes and NFkB, as reported in *Drosophila* (Tsapras et al., 2022).

There are clear examples of nonautophagic processes where atg8ylation plays anti-inflammatory roles. LAP, a manifestation of atg8ylation, has important roles in inflammation and is required for elimination of pathogens (Martinez et al., 2015). LAP deficiency results in development of autoinflammatory, lupus-like syndrome (Martinez et al., 2016). LAP is activated in patients with cirrhosis and protects against hepatic inflammation and fibrosis in mice (Wan et al., 2020). LAP provides immunity against *Streptococcus pneumoniae*, which diminishes as LAP declines with aging (Inomata et al., 2020). In addition to its role in inflammation and related complications, LAP has roles in cancer progression. LAP regulates the production of anti-inflammatory cytokines in tumor-associated macrophages, which promotes immune tolerance in the cancer microenvironment by suppressing T lymphocytes (Cunha et al., 2018).

In summary, atg8ylation, in the context of autophagy or other autophagy-related and unrelated processes, is associated with nearly all key innate immunity systems and, with few exceptions, reduces inflammation. The systems and activities involved in anti-inflammatory activities of atg8ylation and canonical autophagy as one of its outputs engage both regulators of metabolism and key metabolic factories, i.e., mitochondria, with effects on immunometabolism that will be explored in the section on immune cells.

### Antimicrobial functions of atg8ylation and autophagy

Xenophagy (Levine, 2005) is a term often encountered in describing autophagic elimination of intracellular microbes (Gomes and Dikic, 2014; Randow and Youle, 2014). Canonical autophagy or noncanonical atg8ylation processes can protect host cells against a variety of pathogens (Fig. 5 B, box I) including bacteria, viruses, and fungal pathogens. Mechanistic studies in vitro have established that selective autophagy receptors, in particular SLRs, recognize intracellular pathogens or vacuoles containing them when tagged by ubiquitin or galectins and execute their elimination by delivering the pathogens to degradative compartments (Gomes and Dikic, 2014; Randow and Youle, 2014). Some of the microbes studied in depth (Table S1) in this context are *Salmonella*, *Streptococcus*, and *Mycobacterium tuberculosis*. Additional notable examples of bacteria affected by autophagy or noncanonical atg8ylation processes include *Shigella flexneri* and *Listeria*. Besides bacteria, autophagy-related processes affect other microbes including viruses, fungal pathogens, and protozoa such as *Toxoplasma gondii* (Table S1).

The professional intracellular pathogens, such as intracellular bacteria (Engstrom et al., 2019; Laopanupong et al., 2021; Mitchell et al., 2018; Strong et al., 2020; Tan et al., 2018; Zhang et al., 2019b), viruses (Chang et al., 2019; Kumar et al., 2021a; Kyei et al., 2009; Orvedahl et al., 2007; Tiwari et al., 2020), and protozoa (Lopez Corcino et al., 2019), have evolved a multitude of ways to counter or disarm autophagy or components of atg8ylation (Xu et al., 2022; Xu et al., 2019; Fig. 5 B, box II). In extreme cases, highly adapted intracellular pathogens can use autophagy or factors contributing to atg8ylation and autophagy-related processes to support their own growth (Fig. 5 B, box III). This includes *Anaplasma phagocytophilum* (formerly known as *Ehrlichia*, from order *Rickettsiales*; Niu et al., 2012), hepatitis C virus (Lee and Ou, 2021; Twu et al., 2021), influenza A virus (Beale et al., 2014), and SARS-CoV-2 (Hoffmann et al., 2021; Schneider et al., 2021; Twu et al., 2021). Probably the most elegant examples of bacterial defenses come from studies of *Legionella pneumophila* and *Salmonella* (Fig. 5 B, box II). *L. pneumophila* encodes a factor, RavZ, that irreversibly counters atg8ylation by proteolytically removing mATG8s’ C-terminal glycines, thus precluding lipidation (Choy et al., 2012). *Salmonella* SopF acts as an ADP-ribosylating enzyme modifying v-ATPase to block its recruitment of ATG16L1 and inhibit atg8ylation (Xu et al., 2022; Xu et al., 2019). It is important to keep in mind that the majority of bacteria are not equipped to combat autophagy as one of the outputs of atg8ylation, and only a handful of highly adapted intracellular bacteria can persist in host cell’s cytosol. Considering that mitochondria evolved from Rickettsia-like endosymbionts, this presents us with a clear example of a terminal bacterial–host adaptation in coevolution with autophagy (Deretic, 2010; Randow and Youle, 2014; Youle, 2019).

There is a relative dearth of studies of atg8ylation in animal models compared with the wealth of ex vivo studies using cellular models of microbial invasion. The available murine models suggest that xenophagy, even when it can be documented in vivo, is accompanied by an equal or more important action of canonical and noncanonical autophagy processes as atg8ylation outputs in protecting against excessive inflammation and tissue damage (Deretic, 2021; Deretic and Levine, 2018). A hyperinflammatory state can be artificially generated in murine models when genes contributing to autophagy or atg8ylation are inactivated. This paradoxically presents itself as protective against infections in experimental situations (Fig. 5 A, box III). For example, inactivation of key atg8ylation genes and/or components of HyPAS in myeloid cells elevates respiratory tract inflammation and confers resistance to influenza in mice (Lu et al., 2016). Similar genetic maneuvers can be protective against murine herpesvirus reactivation (Park et al., 2016). Likewise, disrupting tissue macrophage quiescence by inactivating genes contributing to HyPAS but not atg8ylation, which normally maintains the anti-inflammatory state, confers resistance to *Listeria* in mice (Wang et al., 2020b). Autophagy or autophagy-related processes are important to prevent cytotoxicity upon stimulation with PAMPs (Levy et al., 2020) or IFN-γ (Orvedahl et al., 2019). Two conclusions can be drawn from these explorations: First, processes associated with autophagy-like phenomena and atg8ylation in principle play anti-inflammatory roles. Second, not all components of what has been classically considered canonical autophagy are needed.

#### Coronaviruses and SARS-CoV-2

The relationship between autophagy and coronaviruses has a relatively extensive history of studies preceding the emergence of SARS-CoV-2 and deserves special attention. Atg8ylation, autophagy, and coronaviruses intersect (Cottam et al., 2011; Cottam et al., 2014; Fung and Liu, 2019; Prentice et al., 2004; Reggiori et al., 2010). Depending on the viral species, autophagic processes have been reported to suppress coronaviruses (Guo et al., 2016; Ko et al., 2017), support their growth (Guo et al., 2017b; Prentice et al., 2004; Zhu et al., 2016), or have no effects (Schneider et al., 2012; Zhao et al., 2007). Both coronaviruses and canonical autophagy involve formation of intracellular double membranes. Coronaviruses remodel cellular membranes and generate protrusion-type viral-replication compartments (VRCs; Strating and van Kuppeveld, 2017), consisting of interconnected double membrane vesicles (DMVs), packets of merged DMVs, plus additional convoluted membranes, generating a compartment for active RNA synthesis complexes secluded away within DMVs (Snijder et al., 2020) to avoid recognition by cytoplasmic PRRs, thus minimizing antiviral type I IFN activation. Some aspects of coronavirus VRCs include morphological features of autophagosomes (e.g., DMVs; Snijder et al., 2006) but are clearly distinct from canonical autophagosomes (Reggiori et al., 2010), although mATG8s (LC3) can be found in the vicinity (Cottam et al., 2014; Reggiori et al., 2010). Unbiased global studies of host factors necessary for successful viral infection have all but ruled out the role of core ATG proteins while identifying a role for VMP1 and TMEM41B proteins (Hoffmann et al., 2021; Schneider et al., 2021). The VMP1 and TMEM41B lipid scramblases (Li et al., 2021) play a general role in transfer of membrane lipids from the ER (Ghanbarpour et al., 2021) and may (independently of their role in autophagy) assist formation of SARS-CoV-2 DMVs (Schneider et al., 2021) harboring active RNA synthesis complexes (Snijder et al., 2020; Fig. 5 B, box III). Class III PI3-kinase is coactivated during DMV formation, and DFCP1 marks PI3P-positive domains involved in either autophagosome formation (Axe et al., 2008) or viral DMVs (Twu et al., 2021). It appears that coronavirus DMVs and canonical autophagosomes compete for membrane sources. In keeping with this, SARS-CoV-2 counteracts canonical autophagosome formation (Kumar et al., 2021a; Fig. 5 B, box II). SARS-CoV-2 nsp6 blocks autophagosomal prophagophore formation by interacting with nearly all components (SIGMAR1, VAMP7, E-SYT2, SERCA2, and TBK1) controlling HyPAS formation (Kumar et al., 2021a).

SARS-CoV-2 additionally interferes with other aspects of autophagosomal and lysosomal pathways. ORF3a affects autophagosome-lysosome fusion (Hayn et al., 2021; Miao et al., 2021), whereas SARS-CoV-2 ORF7 interferes with autolysosomal acidification (Hayn et al., 2021). On the flip side, autophagy factors counter actions of SARS-CoV-2 proteins: ATG9A repairs plasma membrane damage induced by SARS-CoV-2 ORF3a (Claude-Taupin et al., 2021). Adding to these antagonistic relationships between the virus and the host, atg8ylation phenomena may play a role in countering COVID-19 pathogenesis. Cells release exosomes dubbed decoy exosomes or “defensosomes” (Fig. 2 D) that can soak up microbial toxins so that they do not attack cells (Keller et al., 2020). This happens in an ATG16L1-dependent fashion (Keller et al., 2020), and has been extended to the release of ACE2^+^ defensosomes that bind and block SARS-CoV-2 entry (Ching et al., 2021 *Preprint*). Presence of ACE2^+^ exosomes in bronchioalveolar lavages of critically ill COVID-19 patients was reported as being associated with reduced hospitalization times (Ching et al., 2021 *Preprint*). These findings suggest that atg8ylation and variants of secretory autophagy may contribute to antiviral defenses, albeit they can help viral egress in other cases, such as atg8ylation-dependent filamentous influenza A budding from the plasma membrane (Beale et al., 2014).

### Immune cells, atg8ylation, and autophagy

The functions of ATG genes in immune cells have been extensively reviewed (Clarke and Simon, 2019; Deretic, 2021; Deretic et al., 2013; Macian, 2019; Riffelmacher et al., 2018). Since canonical autophagy (a classical downstream effector of atg8ylation) is a metabolic process, autophagy in immune cells manifests itself as an immunometabolic phenomenon (O’Neill et al., 2016), tracking with the known roles of mTOR and AMPK (Fig. 5 C). This is reflected in the reported beneficial effects of metformin, a clinically used AMPK activator, which improves immune responses by enhancing survival of memory CD8^+^ T cells (Bohme et al., 2020) and moderates Th17 responses by CD^+^ T cells (Bharath et al., 2020) in infection, diabetes, and “inflammaging” contexts. Canonical autophagy is believed to support self-renewal and quiescence in immune stem cells, as well as cytoplasmic remodeling during immune cell differentiation (Riffelmacher et al., 2018). Atg8ylation components support neutrophils (Riffelmacher et al., 2017), B1a cells (Clarke et al., 2018; Miller et al., 2008), invariant NKT cells (Salio et al., 2014) and NKT cells (Zhu et al., 2018). They are required for T cell survival and function (Jia et al., 2011; Li et al., 2006; Pua et al., 2009) and for preservation of effector and memory CD8^+^ T cells (Puleston et al., 2014; Xu et al., 2014). Autophagy plays a role in the metabolism of CD8^+^ T cells, affecting their antitumor immunity (DeVorkin et al., 2019). These processes counter T cell anergy (Mocholi et al., 2018), whereas tissue-resident memory CD8^+^ cells and mucosa-associated invariant T cells display high autophagy levels (Swadling et al., 2020). Much of the role of atg8ylation in lymphocyte function is to enhance clearance of virus-infected and cancerous cells while minimizing excessive inflammation and tissue pathology.

The role of atg8ylation processes other than canonical autophagy in immune cells or in cells that are targets of cell-mediated immunity are only beginning to be investigated. This often includes modulation of cell surface receptors. Plasma membrane presence of the cystine transporter SLC7A11 is diminished in pancreatic ductal adenocarcinoma cells knocked down for ATG5 or ATG7 (Mukhopadhyay et al., 2021). A further example of receptor regulation (Heckmann et al., 2019; Ulland et al., 2017) by atg8ylation processes and intersections between atg8ylation, autophagy, inflammation, and metabolism, is TREM2, a surface receptor required for microglial responses and a risk factor in sporadic Alzheimer’s disease (Shi and Holtzman, 2018). An atg8ylation process LANDO promotes recycling of the TREM2 which allows more efficient clearance of Aβ by the microglia and mitigates inflammatory microglial activation (Heckmann et al., 2019; Fig. 5 A, box III). However, TREM2 also plays a role in immunometabolism through control of mTOR and canonical autophagy and maintains microglial metabolic fitness, which fails in TREM2 hypomorphs and is correctable by cyclocreatine supplementation (Ulland et al., 2017). MHC-I downregulation contributes to pancreatic cancer being immunologically cold and recalcitrant to check point inhibitors immunotherapy (Yamamoto et al., 2020; Fig. 5 A, box IV). This downregulation depends on FIP200 and other components of the FIP200 complex, whereas Rubicon seems not to affect it (Yamamoto et al., 2020), thus ruling out LAP-like processes that depend on Rubicon (Heckmann et al., 2019; Martinez et al., 2015). Until recently, it has been very difficult to envision how a single membrane endosome formed during MHC-I endocytosis might result in degradation by canonical autophagy. However, the latest findings (Kumar et al., 2021a) demonstrating that canonical autophagosomes are generated through fusion between endosomes and FIP200^+^ cis-Golgi membranes thus forming the HyPAS pro-phagophore can explain how endocytosed proteins/receptors end up being degraded in canonical autolysosomes. Atg8ylation, in the form of LAP, by tumor-associated macrophages is immunosuppressive, as found in a murine model of melanoma where LAP reduces STING-mediated type I IFN response normally important to spur infiltrating antitumor T cells (Cunha et al., 2018). This aspect seems to be of general import, as enhanced STING-mediated type I IFN responses due to inhibition of atg8ylation or autophagy are beneficial in cancer or cancer treatment models (Poillet-Perez et al., 2020; Yamazaki et al., 2020; Fig. 5 A, box IV). Thus, various proteins at the surface of immune cells or of cells that are their targets are affected by atg8ylation systems with important immunological consequences.

Mitochondrial abundance and their functionality depend on atg8ylation through mitophagy, which is essential for proper function of immune cells, and therefore their participation in clearance of infection or cancer and tissue repair and homeostasis (Li et al., 2019; Nakahira et al., 2011; Sliter et al., 2018). Developmental mitophagy is immunologically important in nonimmune cells because retention of erythroid mitochondria can trigger autoimmune diseases such as lupus (Caielli et al., 2021). Given the evolutionary origins of mitochondria from cytosol invading bacteria, it is not surprising that mitochondria to this day serve as signaling platforms for activation of protective innate immunity in response to bacterial and viral PAMPs. Under pathological conditions, mitochondria themselves become sources of endogenous DAMPs (oxidized mtDNA, dsDNA, dsRNA, cardiolipin, ROS), which stimulate a variety of PRRs and contribute to degenerative and inflammatory states (Youle, 2019). Mitochondria are furthermore the principal contributors to immunometabolism (Mills et al., 2017). This is of particular significance in immune cells whereby switching between oxidative phosphorylation (OXPHOS) and glycolysis has to match or even determines activation levels of T cells, macrophages, dendritic cells (Mills et al., 2017), and as recently shown, neutrophils (Willson et al., 2022; Fig. 5 C). It is well known that glycolysis dominates in proinflammatory classically activated macrophages whereas OXPHOS predominates in alternatively activated macrophages involved in tissue repair (Mills et al., 2017). Even in neutrophils, where mitochondrial electron transport complexes are traditionally believed to have very limited expression, recent studies show that they nevertheless contribute to ROS formation and stabilization of HIF-1α under hypoxic conditions in inflammatory sites (Willson et al., 2022), as during pulmonary complications in covid patients (Ackermann et al., 2021). Mitophagy has been shown to play an important role in immune cell metabolic switch from glycolysis to OXPHOS.

In conclusion, the common themes regarding the role of atg8ylation and autophagy as its thus far best studied output in immune cells include effects on immunometabolism, surface receptor expression, cytoplasmic immune signaling complexes, mitochondrial abundance, and quality control, all with consequences for proper function of immune cells and systems in various tissues and in diverse disease contexts.

## Conclusions and outlook

The expanded concept of atg8ylation as a membrane stress response has many physiological effects based on quality control, metabolism, and roles in immunity. Experimental analyses have revealed that many mammalian ATG genes, including mATG8s, function in processes other than canonical autophagy. The atg8ylation concept—a covalent modification of stressed or remodeling membranes—mirrors ubiquitin modification of proteins. The crossovers between ubiquitylation and atg8ylation exist, as evident from examples of the ubiquitylation of glycolipids such as bacterial LPS when eroding into the mammalian cell cytoplasm (Otten et al., 2021). Conversely, protein atg8ylation can occur (Agrotis et al., 2019; Nguyen et al., 2021).

Atg8ylation participates in an expanding list of processes and includes but is not limited to canonical and noncanonical autophagy as its manifestations. In this view, the noncanonical membrane modifications and remodeling processes such as LAP, LANDO, selective microautophagy, EV/exosome secretion, and secretory autophagy, are easier to systematically organize and understand. The concept of atg8ylation also offers a new perspective on drug development by differentiating atg8ylation from canonical autophagy as targets of interest in different diseases.

The immune functions of canonical and noncanonical autophagy, and the emerging concept of atg8ylation as a membrane stress response, have been summarized herein. They manifest themselves in interactions with microbial pathogens as well as in cancer and as containment of endogenous sources of inflammation. Classically, autophagy is based on digestion of self at times of starvation, organellar malfunction, or elimination of incoming microbes during infection. These functions are conflated in mitophagy, as a product of an evolutionary “truce” between the host and an endosymbiont of bacterial ancestry. Autophagy is likely tirelessly at work against microbial would-be invaders, and, as emphasized here, in protection against endogenous sources of inflammation. Harnessing the full potential of autophagy and atg8ylation in separate and more granular ways may help in protection against current and future pandemics, as well as in treatments of cancer, neurodegenerative, and other diseases.

## Online supplemental material

Table S1 shows examples of intracellular microbes studied as targets for elimination by autophagy.

## Supplementary Material

Table S1 shows examples of intracellular microbes studied as targets for elimination by autophagy.
