# Supplementary material for: A guide to membrane atg8ylation and autophagy with reflections on immunity
Source: J Cell Biol. 2022 Jun 14;221(7):e202203083. doi: 10.1083/jcb.202203083 (PMC9202678; doi:10.1083/jcb.202203083)
Supplement: Table S1 — shows examples of intracellular microbes studied as targets for elimination by autophagy. [file JCB_202203083_TableS1.docx]

**Table S1.**

Examples of intracellular microbes studied as targets for elimination by autophagy

| **Microbes** | **Genus or species** | **References** |
| --- | --- | --- |
|  |  |  |
| Bacteria | Salmonella | (Birmingham et al., 2006; Cemma and Brumell, 2012; Dooley et al., 2014; Jia et al., 2009; Kageyama et al., 2011; Millarte et al., 2022; Ravenhill et al., 2019; Thurston et al., 2009; Thurston et al., 2012; Tumbarello et al., 2015; von Muhlinen et al., 2012; Wild et al., 2011; Xu et al., 2022; Xu et al., 2019) |
|  | *Streptococcus pyogenes*  (when eroding into the host cells) | (Lin et al., 2020; Minowa-Nozawa et al., 2017; Nakagawa et al., 2004; Nozawa et al., 2020; Wang et al., 2020a) |
|  | *Mycobacterium tuberculosis* | (Bryk et al., 2020; Budzik et al., 2020; Castillo et al., 2012; Chai et al., 2019; Chauhan et al., 2016; Franco et al., 2017; Giraud-Gatineau et al., 2020; Gutierrez et al., 2004; Intemann et al., 2009; Jia et al., 2018; Jia et al., 2020a; Kimmey et al., 2015; Laopanupong et al., 2021; Manzanillo et al., 2013; Pahari et al., 2020; Paik et al., 2019; Palma et al., 2021; Ponpuak et al., 2010; Singh et al., 2006; Tur et al., 2020; Watson et al., 2012; Xie et al., 2017; Zhang et al., 2019a) |
|  | *Shigella flexneri* | (Maculins et al., 2021; Mostowy et al., 2011; Noad et al., 2017; Ogawa et al., 2005) |
|  | *Listeria monocytogenes* | (Gluschko et al., 2018; Mitchell et al., 2018; Tur et al., 2020; Wang et al., 2020b; Yano et al., 2008) |
| Viruses | Various | (Jounai et al., 2007; Liang et al., 1998; Liu et al., 2021; Miyakawa et al., 2022; Montespan et al., 2017; Orvedahl et al., 2010; Sumpter et al., 2016; Wang et al., 2021) |
| Fungi | Various | (Kanayama and Shinohara, 2016; Ligeon et al., 2021; Nicola et al., 2012; Ohman et al., 2014) |
| Protozoa | *Toxoplasma gondii* | (Andrade et al., 2006; Bhushan et al., 2020; Burger et al., 2018; Sasai et al., 2017; Selleck et al., 2015). |
